# Bonding Effectiveness of Two Adhesive Luting Cements to Glass Fiber Posts: Pull-Out Evaluation of Three Different Post Surface Conditioning Methods

**DOI:** 10.1155/2014/148571

**Published:** 2014-06-02

**Authors:** Lorenzo Graiff, Laura Rasera, Marco Calabrese, Paolo Vigolo

**Affiliations:** ^1^Department of Restorative Dentistry, Institute of Clinical Dentistry, University of Padova, Via Venezia, 90, 35100 Padova, Italy; ^2^Private Practice, Via Vivaldi 13, Farra di Soligo, 31010 Treviso, Italy; ^3^Department of Clinical Odontostomatology, Institute of Clinical Dentistry, University of Padova, Via Venezia, 90, 35100 Padova, Italy

## Abstract

The purpose of this study was to evaluate the bond strength at the post/resin-cement interface with 3 different surface treatments of glass fiber posts and with 2 different luting resin cements. Sixty glass fiber posts (RelyX Fiber Post) were randomly divided into 3 groups (*n* = 20) and were luted with a dual-polymerizing self-adhesive universal resin cement (RelyX Unicem) and with a dual-polymerizing resin cement (RelyX ARC). This was carried out in association with a dual-polymerizing adhesive (Scotchbond Multi-Purpose Plus) in simulated plexiglass root canals after receiving three different pretreatment procedures. A pull-out test was performed on each sample to measure bond strengths. Data were analyzed with two-way ANOVA. Two samples from each group were processed for SEM observations in order to investigate the morphologic aspect of the post/cement interface. Both resin cements demonstrated significant different bond strength values (*P* < 0.0001). The surface treatment result was also statistically significant (*P* = 0.0465). SEM examination showed a modification of the post surface after pretreatment with methyl methacrylate. The dual-polymerizing self-adhesive universal resin cement achieved higher MPa bond strength values. The use of methyl methacrylate as a surface treatment of glass fiber posts provided a significant increase in bond strengths between the posts and both luting materials.

## 1. Introduction

For the restoration of endodontically treated teeth, the use of fiber-posts luted with resin cement and combined with composite core build-up materials is becoming very frequent. Since 1990 many new fiber posts and resin adhesive luting systems have been made available by industry and numerous researches revealed their successful clinical results when joined to adhesive/resin systems, due to their superior retention values and behavior under mechanical stress [1–3]. Since fiber-reinforced posts have a modulus of elasticity (E) similar to that of dentin (resp., 18–22 GPa and 18 GPa), they produce a stress field similar to that of natural teeth, thereby reducing the risk of root fracture [4–7].

The selection of the composite plays an important role, not only for the clinical success of a post/core restoration but also for the quality of the post/core interface, where different materials are in close contact [8]. Many studies focused particularly on the possibility of improving adhesion at the fiber post-composite interface through various treatments of post surface [9–12]. Surface treatments are common methods for improving the general adhesion properties of a material by facilitating chemical and micromechanical retention. To improve the bonding of resin cements to fiber posts, several treatments of the post surface have been suggested, such as etching with hydrofluoric acid, the use of hydrogen peroxide or potassium permanganate, the airborne-particle abrasion with aluminum oxide or silica particles, and the application of a silane coupling agent [13–19]. Resin-based adhesive luting materials are widely used for the fixation of posts, and currently all the resin cements are based upon the use of either an etch-and-rinse or of a self-etch adhesive, along with a low-viscosity resin composite. This multistep application technique is complex and somewhat technique sensitive and consequently may compromise bonding effectiveness. Recently, a new resin-based cement (RelyX Unicem Cement, 3 M ESPE, Seefeld, Germany) has combined the use of adhesive and cement in one single application, eliminating the need for pretreatment of both tooth and restoration. The adhesive proprieties are claimed by the manufacturer to be based upon acid monomers that demineralize and infiltrate the tooth substrate, resulting in micromechanical retention. Secondary reactions have been suggested to provide chemical adhesion to hydroxyapatite, a feature currently only proven for glass-ionomers. Moreover, according to manufacturer data, RelyX Unicem Cement should adhere to RelyX Fiber Posts (3 M ESPE) in 3 ways: mechanical interlocking, covalent bonds, and hydrogen bonds. A recent systematic review of the role of resin cement on bond strength of glass-fiber posts seems to suggest that the use of self-adhesive resin cement, especially with Relyx Unicem, could improve the retention of glass fiber post in root canals [20].

The purpose of this in vitro study was to evaluate the influence of different post-surface treatments on the interfacial bond strength between glass fiber-reinforced posts and 2 different resin luting cements. In this study an experimental model to simulate an endodontic channel has been used: this permitted the analysis of only a particular aspect of the adhesion of the posts to the root canals, the post-cement interface, excluding interference of the cement-dentin interface [17, 21]. The morphological aspects of the fibers and the post-surface characteristics following the different pretreatments were also observed using scanning electron microscopy (SEM). Pull-out tests were performed on the samples to measure the bond strength at the post/resin cement interface. The null hypothesis test was to demonstrate that the type of resin cement used for post cementation has no influence on the post-cement interfacial bond strength.

## 2. Materials and Methods

### 2.1. Study Design and Selected Products

A simulated root canal (a plexiglass mold equipped with an artificial tapered post space, industrially prepared (3 M ESPE) with a coronal diameter of 1.80 mm, an apical diameter of 1.30 mm, and a length of 15 mm) was used to exclude interference of the cement dentin interface as illustrated in previous articles [17, 21].

Sixty glass fiber posts (RelyX Fiber Post, 3 M ESPE), size number 1, with a diameter of 0.70 mm at the apical post end and a diameter of 1.30 mm at the coronal post end, were used in this study. All the materials used in this study are presented in Table 1. To evaluate the effects of different surface treatments on bond strength, all posts were divided into 3 groups (*n* = 20) and received different surface treatments.

- Group 1: all posts were brushed with ethanol (95%) for 30 seconds and dried with air for 10 seconds (control).
- Group 2: all posts were pretreated with methyl methacrylate (Pro Base Cold Monomer, Ivoclar Vivadent, Schaan, Liechtenstein) for 1 minute and dried with air for 10 seconds.
- Group 3: all posts were pretreated with methyl methacrylate (Pro Base Cold Monomer) for 1 minute and dried with air for 10 seconds and tribochemically coated (CoJet, 3 M ESPE).

The pretreatment with methyl methacrylate was effected by immersing every post in a dappen dish for 1 minute at room temperature; after 1 minute the posts were gently air-dried for 10 seconds. In group 3, after the pretreatment with methyl methacrylate, the post was tribochemically coated. Silica-coated alumina particles (30 *μ*m diameter) were blasted onto the post surface for 20 seconds at 2.8-bar pressure from a distance of approximately 50 mm.

Every group was divided into 2 subgroups, A and B. In subgroups A, the posts were luted with a dual-polymerizing self-adhesive universal resin cement (RelyX Unicem, 3 M ESPE); in subgroups B, the posts were luted with a dual-polymerizing resin cement (RelyX ARC, 3 M ESPE). In all subgroups B, Scotchbond Multi-Purpose Plus (3 M ESPE) was used for the treatment of posts surface, according to the manufacturer's indications.

The sample size of each subgroup was determined through a power analysis as follows [22]: for the test, a minimum detectable difference among means of 5 MPa was needed. The required probability in order to detect it was set to 0.98 for the material factor and to 0.80 for the pretreatment factor. The resulting sample size was 10 for each material pretreatment combination.

### 2.2. Specimen Preparation and Pull-Out Test

All specimens were prepared by the same investigator to ensure standardization. In every plexiglass block, the post space was roughened with a small round stainless-steel bur to create small undercuts for improving cement retention: in this way an early dislodgment of the cement from the plexiglass during pull-out test could be avoided. In the subgroups A, the self-adhesive universal cement RelyX Unicem was used, in accordance with the manufacturer's instructions. RelyX Unicem was processed in a capsule system (Aplicap, 3 M ESPE), activated with the Activator Aplicap, and mechanically triturated with a mixing machine (Silamat, Ivoclar Vivadent) for 15 seconds as recommended by the manufacturer; the cement was then applied into the artificial post spaces with light pressure and rotation using the correspondent Aplicap Elongation Tip (3 M ESPE) in order to apply cement in the canal from bottom to top. Posts were positioned into the artificial root canals immediately after the insertion of the resin cement, and photopolymerization with a LED curing unit (Bluephase, Ivoclar Vivadent AG. FL, 9494 serial number 1537528) was performed from the top of plexiglass endo-block for 40 seconds.

Light intensity was measured with a visible curing light meter (Cure Rite, Dentsply Caulk, Model number 644726, Serial number 7239, Milford, DE 19963, USA), and the output was 1370 mW/cm^2^. In the subgroups B, where the resin cement RelyX ARC was used, every post surface received, in addition to the surface pretreatment, a single coat of catalyst (Scotchbond Multi-Purpose Plus Activator 3.5; 3 M ESPE) applied with a microbrush and gently air-dried for 5 seconds. The dual-polymerizing resin cement (RelyX ARC, 3 M ESPE) was placed into the artificial root canals with the aid of a spiral drill (Lentulo, Dentsply Maillefer, 1338, Ballaigues, Switzerland), and the posts were placed into the canals with light pressure and rotation. Excess luting material was removed with a disposable microbrush; then photoactivation was performed for 40 seconds (Bluephase, Ivoclar Vivadent).

**Figure 1:**
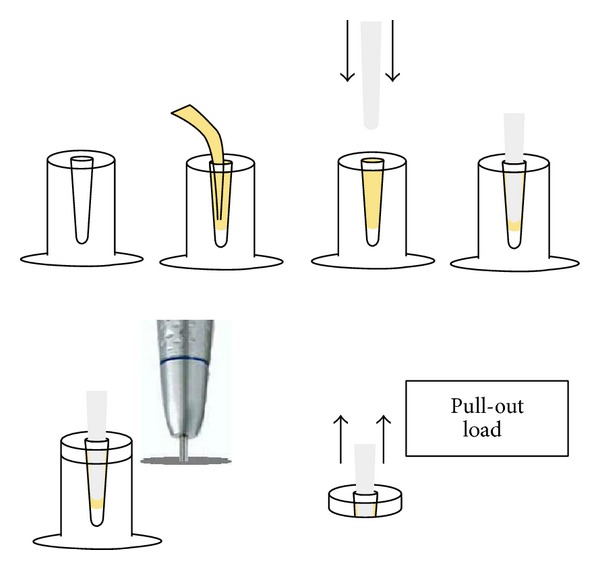
Schematic drawing of preparation of specimens for pull-out test.

**Figure 2:**
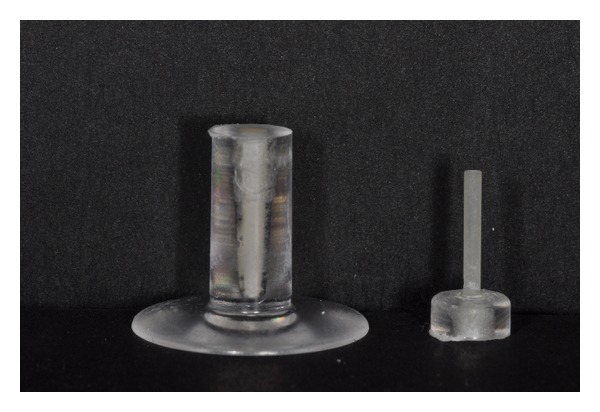
Sample after cutting slice for pull-out test.

Every post was luted into the artificial post space to a length of 10 mm using a rubber ring as a stop. The plexiglass blocks were sectioned to obtain 3 mm thick slices in order to reduce the necessary force to debond posts from the cement during the pull-out test (Figure 1). No pre-test failures occurred during cutting and testing procedures. For every sample, only one section was obtained to be used for the pull-out test, for a total of 60 specimens (Figure 2).

The pull-out test was performed along the long axis of the post at a cross-head speed of 1.00 mm/min. The post was loaded (pull-out) until its extrusion from the plexiglass block disks by means of a universal testing machine (Erichsen 476, ERICHSEN GmbH & Co. KG, Hemer, Germany). For the pull-out test, a stainless steel device suitable for the testing machine was realized in order to be able to bond firmly onto the post on its upper side; the force required to dislodge each post was then recorded in Newtons.

Bond strength was expressed in MPa, dividing the load at failure (newtons) by the bonding surface area. As the bonded interface was the lateral surface of the post, 3 mm in height, its area was calculated using the formula of a conical frustrum: *π*(*R*_1_ + *RB*_2_)√(*R*_1_−*R*_2_)^2^+*h* ^2^ [23].

After testing, failure modes were evaluated with a stereomicroscope (Leica Microsystems GmbH Wetzlar, Germany) at 20x magnification and recorded as adhesive, cohesive, and mixed.

The types of fracture were classified in 3 categories:

1. adhesive, at the post/composite interface (no resin cement visible around the post),
2. cohesive within the composite,
3. mixed with resin cement covering the post surface between 0 and 50%.

Data were analyzed with two-way ANOVA since for this dataset underlying assumptions are completely fulfilled.

### 2.3. Scanning Electron Microscopy (SEM)

Two specimens from each group were examined with a SEM (JSM 6490 JEOL Ltd. Akishima, Tokyo, Japan) in order to evaluate the characteristics of the post surface after various chemical treatments and the post/cement interface after the pull-out test. Each specimen was sputter-coated with gold-palladium and examined with a SEM at different magnifications.

## 3. Results 

### 3.1. Pull-Out Test

Data from the pull-out test are presented in Table 2. The results obtained from the testing machine (N) were divided by the previously calculated bonding surface area (mm^2^). Debonding stress values were converted to megapascals (MPa).

### 3.2. Statistical Analysis

**Figure 3:**
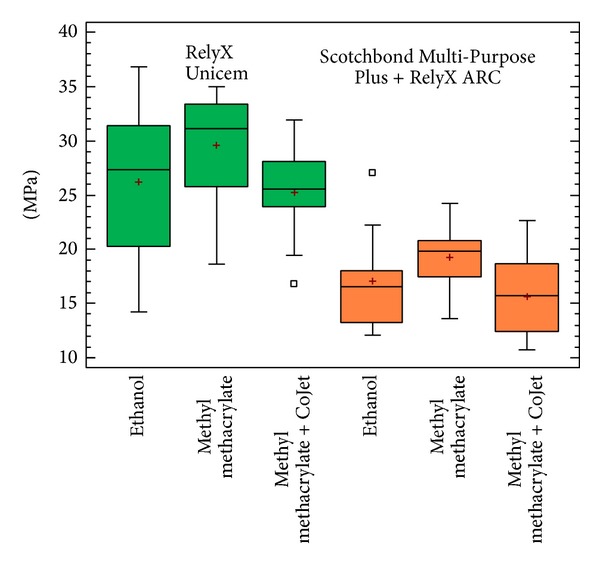
Box and whisker plots for each sample.

**Figure 4:**
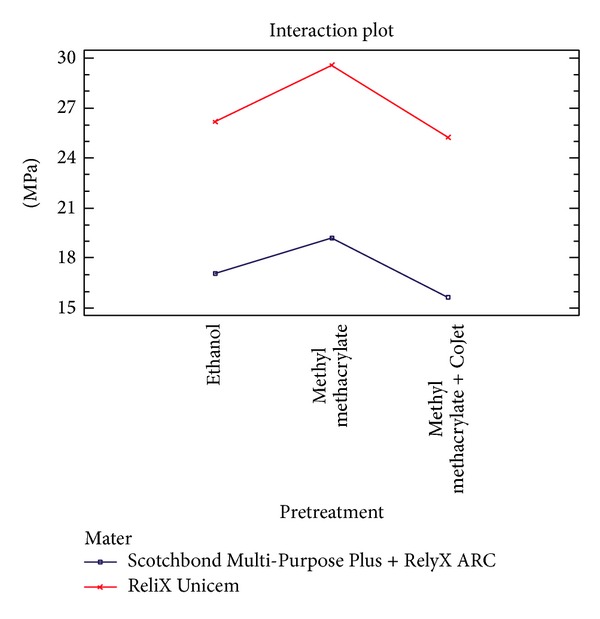
Plot of interaction effect between pretreatment and cement material.

In Figure 3, the comparative box and whisker plots for each sample are shown. In order to detect significant differences among groups statistically, a two-way analysis of variance (ANOVA) was applied with pull-out bond strength in MPa as the dependent variable, with luting material and surface pretreatment procedures as factors. The interaction between the two factors was included in the analysis. The statistical analysis was processed by “R” software (2.12.2 Free Software Foundation's GNU general Public License). Statistical analysis revealed that the interaction between the surface pretreatment and the post cementation did not have any significant effect (*P* > 0.9). The effect of the surface pretreatment was the same, regardless of the type of resin cement used (and, conversely, the type of cement effect was the same whichever pretreatment was applied: see Figure 4). This meant that the optimal pretreatment and the optimal cement material could be chosen independently of each other. In detail, RelyX Unicem proved to be superior compared to Scotchbond Multi-Purpose Plus and RelyX ARC (*P* < 0.0001), and the pretreatment resulted also as a significant factor (*P* = 0.0465); in particular, methylmetacrylate post conditioning gave higher bond strength than the other two treatments used which were equivalent.

**Figure 5:**
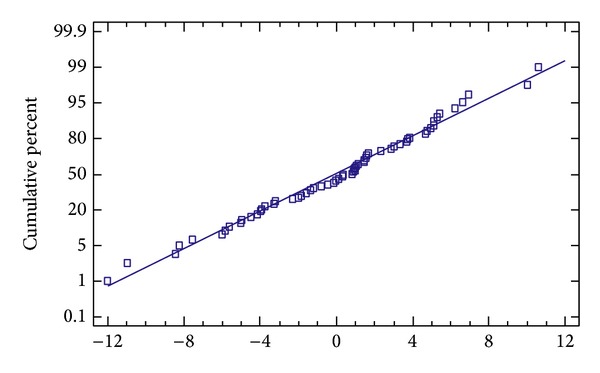
Normal probability plot of ANOVA residuals.

It should be noted that the assumptions underlying the ANOVA tests applied were completely fulfilled for this dataset. In Figure 5 a normal probability plot of residuals of the ANOVA model is shown; since only minor departures of points from the theoretical line can be observed, it should be concluded that residuals agree with a Gaussian distribution assumption. Moreover, in order to check the assumption of constant variance among the different groups, Levene's test was performed. The result of this test leads to the conclusion that there was no statistically significant difference amongst the standard deviations (*P* = 0.1253).

With regard to the type of failure (Table 3), it was observed that it was independent of pretreatment (Pearson's chi-squared test *χ* ^2^ = 0.987, *P* = 0.6105). Conversely, the null hypothesis of independence between resin cement and type of failure (*χ* ^2^ = 37.297, *P* < 0.01) could not be accepted. When RelyX ARC was used as the resin cement, the failures were predominantly adhesive between post and cement. When RelyX Unicem was used, the failures were mixed with portions of resin cement visible around the post. No cohesive fractures were revealed.

### 3.3. SEM Evaluation

**Figure 6:**
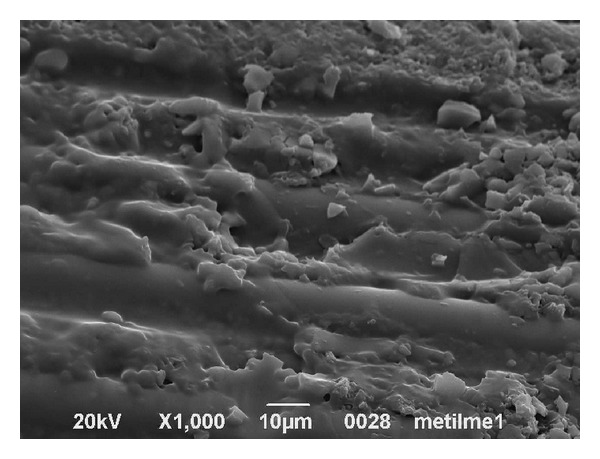
SEM image of the post surface after treatment with Methyl methacrylate for 1 min (1000x).

**Figure 7:**
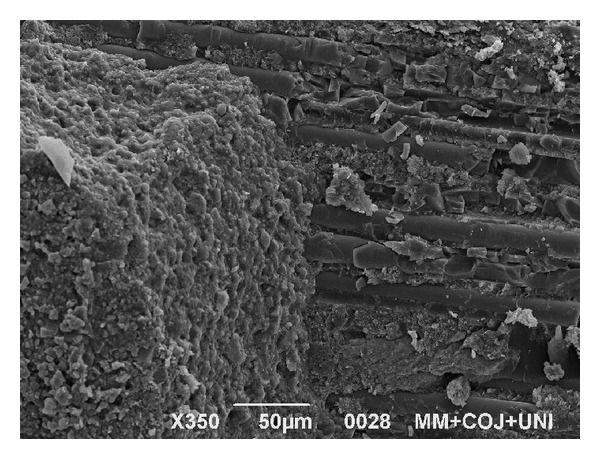
SEM image of the post surface after pull-out test (sample in group 2). A portion of RelyX Unicem on the post surface (350x) is visible.

SEM evaluation revealed that the post surface morphology was modified following treatment with methyl methacrylate and that this treatment produced a change in the ultrastructure of the post surface. In Figure 6 a particular of a post sample surface (group 2A) is shown: it was possible to observe a partial dissolution of the organic matrix of the post. Exposed fibers did not appear to be damaged by the action of methyl methacrylate, and no defects or fractures were evident on their surfaces. SEM evaluation of the specimens after the pull-out test revealed the presence of a portion of resin cement on the post surface, especially when RelyX Unicem was used (Figure 7). This is in accordance with the presence of a great number of mixed fractures visible on the post surface of the specimens in which RelyX Unicem was used. In the groups where Scotchbond Multi-Purpose and RelyX ARC were applied, adhesive fractures were observed, both on airborne-particle abraded and control posts, without any difference in occurrence and morphological appearance.

## 4. Discussion

As recently described in scientific literature, also in this study, an experimental model to simulate an endodontic channel has been used [17, 21]: this permitted the analysis of only one particular aspect of the adhesion of the posts to the root canals, the post-cement interface, excluding interference of the cement-dentin interface [17, 21]. The retention of the cement onto the post depends on the strength of the chemical and micromechanical interaction between a fiber-reinforced material and resin cement. In this study, a simulated root canal was used to exclude interference of the cement dentin interface: if extracted human teeth had been used, the effect would probably not have been detected due to premature debonding based on lower bond strength between post and dentin. The use of extracted teeth would have added many more variables: the method of detersion of dentin, the type and timing of dentin pretreatment, and the type of dentin (dentin of a vital tooth or of a previously endodontically treated tooth).

In this investigation, the adhesion of 2 types of resin cements (a dual-polymerizing self-adhesive universal resin cement and a dual-polymerizing resin cement combined with a dual-polymerizing enamel-dentine adhesive) to a glass fiber post was assessed with the pull-out test. According to the results of this study, the RelyX Unicem system seems to be the most appropriate method for post adhesion, and it recorded a significantly higher bond strength, since a statistical difference was found between the two different resin cements used. The pull-out test showed that the bond strength in the specimens prepared using methyl methacrylate as surface pretreatment was superior to that recorded in the other specimens using the same resin cement. According to the results of this study, the RelyX Unicem system seems to be the most appropriate method for post adhesion and recorded a significantly higher bond strength, since a statistical difference was found between the two different resin cements used. The null hypothesis was rejected.

Adhesive post restorations rely on the strengths of the bonds established at different interfaces for their retention: in fact, the interface between root dentin and resin cement has been investigated in several studies involving bond strength tests and microscopic investigations [24–26]. For a successful adhesion of the resin cement, it is necessary to establish a strong bond between resin and post as well as between resin and dentine. If bonding at these interfaces is poor, debonding and/or fracture of the post and core will occur. Therefore, good adhesion of these interfaces is an important factor for a successful fiber post restoration [17].

In order to improve the bond strength between the post and the resin cement, many surface pretreatment procedures for posts that involve the use of mechanical or chemical agents have been investigated [9–26]. Mechanical treatment is aimed at roughening the post surface, thus enhancing mechanical interlocking between post and resin cement. The use of chemical surface treatment procedures affects the interfacial bond strength between fiber posts and core build-up materials. In a recent study, the use of hydrogen peroxide for pretreatment significantly enhanced the interfacial bond strength between fiber post and resins materials used for core build up [19]. Silane coupling agents have been utilized in dentistry since the advent of glass-reinforced resin-based materials. According to recently published data, treating the post surface with a silane coupling agent is advisable in order to enhance the adhesion of the resin cement used for luting [11].

Besides silanization, also post airborne-particle abrasion and the combination of this with silane coating were found to significantly increase the bond strength of resin cement to glass fiber posts [16]. In group 3 of this study CoJet was used after post conditioning with methyl methacrylate, but this post surface treatment did not produce a statistically significant increase in the bond strength for both luting cements used.

From the results of the pull-out test, it is possible to consider the majority of the failures in this study as mixed failures, in which there was some resin cement still visibly covering the post surface. This is connected with the high bond strength between the two components investigated.

SEM analysis showed significant changes of the post surface after methyl methacrylate conditioning pretreatment, but few irregularities were visible. It seems that the epoxy resinous matrix is partially dissolved on the post's surface, presumably favoring a greater interaction with the resinous cements. It is possible that the adhesion mechanism between the resin cement and the post surface treated with methyl methacrylate may be mainly chemical and not micromechanical. Further investigation could either confirm this hypothesis or discount it. In all the groups in which RelyX ARC was used, numerous adhesive fractures were observed at the resin cement/post interface, although they were also observed in those groups in which methyl methacrylate was used as pretreatment.

When RelyX Unicem was used, it was possible to recognize a great number of mixed fractures. A reason for these different results may be connected to the chemical bond between the parts and in addition, probably to the micromechanical interlocking as indicated by the manufacturer. Moreover, it could be possible that the application of methyl methacrylate as a pretreatment on the post surface may increase the chemical adhesion of the two resinous components.

At the SEM evaluation, the airborne-particle abraded specimens did not appear to be very rough and the CoJet sand particles seemed to be immersed in the resin matrix. Those particles did not contribute to the micromechanical adhesion and this fact can support the decrease of the bond strength between the fiber post and the resin cement due to the reduction of the micromechanical interlocking for the resin cement.

Many previous studies have demonstrated the role of airborne-particle abrasion in increasing the strength of adhesion of resin cement to fiber posts [13, 18, 26], and it was demonstrated that an increment of the adhesion force was essentially due to a type of micromechanical adhesion that was developed between the two surfaces. In this study, the results showed that the use of CoJet after methyl methacrylate post conditioning did not produce a statistically significant increase in the bond strength.

Surface treatments with only methyl methacrylate increased the retention of the glass fiber posts for both RelyX Unicem and RelyX ARC in association with Scotch Bond MP, where methyl methacrylate and CoJet were used for treating post surfaces and the bond strength decreased. Although airborne-particle abrasion may give an increase in microtensile bond strength to glass fiber posts, its effects after methyl methacrylate post conditioning should be further investigated and new research is necessary in order to agree on the optimal particle size, distance, pressure, and time of application. Future studies should also demonstrate the type of bond that develops on those fiber posts (RelyX Fiber Post) which have been pretreated with methyl methacrylate and luted with RelyX Unicem. The main goal of fiber-reinforced composite material is to achieve a tough bond between the various components so as to get a unique material inside the root canal with improved performance: in this way it is possible to create a “monoblock” between the tooth and the restorative material.

## 5. Conclusions

Within the limitations of this in vitro study, the following conclusions were made: the dual-polymerizing self-adhesive universal resin cement achieved higher MPa values than the dual-polymerizing resin cement combined with a dual-polymerizing bonding system (*P* < 0.0001). The use of methyl methacrylate as a pretreatment agent provided an increased bond strength between glass fiber posts and both resin cements used; the association of methyl methacrylate and airborne-particle abrasion as the surface treatment did not improve the bond strength between the glass fiber posts and the resin cement.

## Clinical Relevance

The use of methyl methacrylate for 1 minute as a surface treatment of glass fiber posts increased the pull-out bond strengths for both test materials used in this study.

## Figures and Tables

**Table 1:** List of materials used in this study.

Materials	Manufacturer	Type	Batch number	Chemical composition*
RelyX Fiber Post size number 1	3M ESPE, Seefeld, Germany	Glass fibers post	56861	60–70% (by weight) of glass fibers which are embedded in a cured epoxy-resin matrix containing zirconia filler
RelyX Unicem	3M ESPE, Seefeld, Germany	Dual-polymerizing self-adhesive universal resin cement	271595	Phosphoric acid methacrylates, dimethacrylates, inorganic fillers (72 wt. %), fumed silica, and initiators
RelyX ARC	3M ESPE, St. Paul, USA	Dual-polymerizing resin cement	3415A1	BISGMA, TEGDMA, and zirconia/silica filler (67.5 wt. %)
ProBase Cold Monomer	Ivoclar Vivadent AG, Schaan, Liechtenstein	Methyl methacrylate	J11291	90–95% methyl methacrylate <5% butandiole dimethacrylate
Scotchbond Multi-Purpose Plus	3M ESPE, St. Paul, Minnesota, USA	Dual-polymerizing enamel-dentinal adhesive	7546 7542 7547	**Activator 1,5:** ethyl alcohol, benzene sulfinic acid, and sodium salt; **primer 2:** water, hema copolymer of acrylic, and itaconic acids; and **catalyst3,5**: BISGMA, HEMA, and benzoyl peroxide
CoJet Sand	3M ESPE, St. Paul, Minnesota, USA	Silicatized sand (particle size 30 µm)	239732	Aluminium oxide >97 silica dioxide <3

*Information from the manufacturers.

**Table 2:** Mean and SD values of the pull-out test.

Groups	Surface treatments and materials used	Mean (MPa)	SD (MPa)
1A	Ethanol and ReliX Unicem	26.18	7.17
2A	Methyl methacrylate and ReliX Unicem	29.55	5.74
3A	Methyl methacrylate, CoJet, and ReliX Unicem	25.25	4.53
1B	Ethanol, Scotchbond Multi-Purpose Plus, and RelyX ARC	17.10	4.64
2B	Methyl methacrylate, Scotchbond Multi-Purpose Plus, and RelyX ARC	19.22	3.13
3B	Methyl methacrylate, CoJet, Scotchbond Multi-Purpose Plus, and RelyX ARC	15.65	3.75

**Table 3:** Type of failure.

Pretreatment	Resin cement	Adhesive	Cohesive	Mixed
Ethanol	RelyX Unicem	0	0	10
	Scotchbond Multi-Purpose Plus and RelyX ARC	8	0	2
Methyl methacrylate	RelyX Unicem	0	0	10
	Scotchbond Multi-Purpose Plus and RelyX ARC	6	0	4
Methyl methacrylaten and CoJet	RelyX Unicem	0	0	10
	Scotchbond Multi-Purpose Plus and RelyX ARC	9	0	1

Total		23	0	37
